# Short-course blinatumomab for refractory/relapse precursor B acute lymphoblastic leukemia in children

**DOI:** 10.3389/fped.2023.1187607

**Published:** 2023-08-04

**Authors:** Jiao Xie, Suxiang Liu, Ming Zhou, Yi Wang, Hailong He, Peifang Xiao, Shaoyan Hu, Jun Lu

**Affiliations:** Department of Hematology and Oncology, Children’s Hospital of Soochow University, Suzhou, China

**Keywords:** short-course, blinatumomab, acute lymphoblastic leukemia, hematopoietic stem cell transplantation, children, refractory/relapse

## Abstract

**Objective:**

To evaluate the clinical efficacy and safety of a short course of blinatumomab in children with refractory or relapsed precursor B-cell acute lymphoblastic leukemia (R/R-BCP-ALL).

**Methods:**

The clinical data of 33 R/R BCP-ALL children aged 0–18 years who underwent a short course of blinatumomab (14 days) between August 2021 and November 2022 were retrospectively collected and analyzed.

**Results:**

Among 33 patients with BCP-ALL, 26 achieved complete remission (CR), with a total remission rate of 78.8% (26/33). The duration of remission was approximately 14 days. Of the 7 children without CR, 5 were still in remission at 28 days. In 11 patients with refractory disease and 22 with recurrence, the remission rates were 90.9% (10/11) and 72.7% (16/22), respectively. The overall survival (OS) rates of the 26 patients with CR and seven patients without CR were 96.1% and 57.1% (*p* = 0.002), respectively, and the disease-free survival (DFS) rates were 96.1% and 42.9% (*p* < 0.001), respectively. Among the 26 patients with CR, 15 underwent bridging hematopoietic stem cell transplantation (HSCT) and 11 did not receive HSCT; with OS rates of 93.3% and 100% (*p* = 0.40) and DFS rates of 93.3% and 100% (*p* = 0.400), respectively. The OS for all patients was 87.9% (29/33) and the DFS was 84.8% (28/33). There were 18 cases (54.5%) of cytokine release syndrome (CRS), 2 cases (6.1%) of severe CRS (all grade 3), 1 case (3.0%) of immune effector cell-associated neurotoxicity syndrome (ICANS), 0 cases (0%) of ICANS ≥ grade 3, and no deaths caused by treatment.

**Conclusions:**

Short-term follow-up revealed a high R/R BCP-ALL remission rate in children treated with a short course of blinatumomab. The toxicity was low and controllable. No significant short-term survival benefits were observed after bridging HSCT with blinatumomab. In developing countries, a short course of blinatumomab can achieve satisfactory outcomes, while reducing household costs and saving medical resources.

## 1. Introduction

Acute lymphoblastic leukemia (ALL) is the most common childhood malignancy, accounting for 25% of all childhood cancers and 75% of all newly diagnosed acute leukemias (1). Advances in the diagnosis and treatment of childhood ALL have been achieved in recent decades. The current 5-year survival rate is 80%–90%, although 10%–20% of cases remain refractory or relapsed and conventional chemotherapy is less effective in these patients (2). In recent years, immunotherapy has brought new hope to children with refractory or relapsed precursor B-cell (R/R BCP) ALL. As a bispecific antibody with a relative molecular mass of 55 kDa (3), blinatumomab mainly kills tumor cells by targeting CD19-positive tumor cells and CD3-positive T cells, forming tight target cell-cell contacts (4), and releasing interleukins, interferons, tumor necrosis factor, perforin, and granzymes (5, 6). Because the half-life of blinatumomab is only 2 h, the recommended mode of administration is continuous 24-hour intravenous infusion (7, 8). Data indicate that the bone marrow remission rate among children with R/R BCP-ALL treated with blinatumomab is approximately 60% (9). Furthermore, in an open-label, single-arm, confirmatory study (BLAST; MCT103-203; NCT01207388) trial, 78% of children achieved bone marrow remission after treatment with blinatumomab (10). Based on its unique mechanism of action, tolerable toxicity, and high response rate, blinatumomab was approved by the US Food and Drug Administration (FDA) in 2017 for the treatment of pediatric R/R BCP-ALL (11). In this study, we retrospectively collected and analyzed the clinical data of 33 children who underwent a short course of blinatumomab for R/R BCP-ALL to assess the clinical efficacy and safety of blinatumomab in pediatric R/R BCP-ALL.

## 2. Materials and methods

### 2.1. Enrolled patients

Thirty-three children with R/R BCP-ALL treated with a short-course of blinatumomab in the Department of Hematology at the Children's Hospital of Soochow University between August 2021 and November 2022 were included in this study. The study population included 21 male and 11 female patients, with a mean age of 7.6 ± 3.7 years. Written informed consent was obtained from the children's guardians, and the study was approved by the Medical Ethics Committee of the Children's Hospital of Soochow University (approval number: ChiCTR2200064906).

The inclusion criteria were as follows: (1) age 0–18 years and (2) refractory/relapsed BCP-ALL (12). Refractory disease was defined as the absence of remission after undergoing at least two induction chemotherapy treatments with no response to targeted drugs or targeted drug therapy. Relapse included morphologic relapse, defined as the reappearance of leukemic cells in the peripheral blood of patients who achieved complete remission (CR), the presence of >5% naïve cells in the bone marrow or the occurrence of new pathologic hematopoiesis, the appearance of leukemia cells outside the marrow, and molecular and/or genetic relapse, defined as the achievement of CR at the cytogenetic or molecular level, followed by the development of cytogenetic or molecular abnormalities. (3) Leukemic cells with a CD19 marker on the surface and (4) written informed consent from the children's guardians were provided for participation in the study. The following exclusion criteria were applied: (1) critical illness, shock, or requirement for mechanical ventilation support; (2) no CD19 marker on the surface of leukemic cells; (3) interruption of treatment for various reasons; and (4) severe psychiatric or neurological disorders or extramedullary relapse.

### 2.2. Treatment strategy

**Figure 1:**
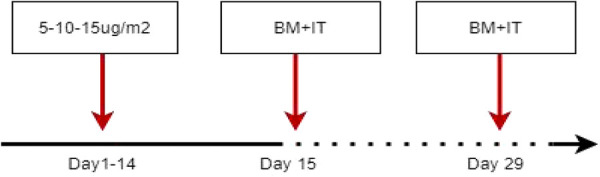
Flow chart of blinatumomab usage. BM, bone marrow; IT, intrathecal; DEX, dexamethasone.

Before the short course of blinatumomab, the patient was treated with dexamethasone (5 μg/m^2^ for 2–3 days), and the dose was gradually increased as follows: 5 μg/m^2^ for 2 days, 10 μg/m^2^ for 2 days, and 15 μg/m^2^ for 10 days. In the event of side effects or signs of toxicity, adjustments were made to the treatment plan according to the manufacturer's instructions. A bone marrow assessment was performed approximately 15 days after the commencement of treatment. If CR was not achieved, blinatumomab was administered at a dose of 15 μg/m^2^ until day 28, and bone marrow assessment was performed again on day 29 [Figure 1]. Hospitalization was required throughout the course of treatment, and the patients were also administered one or two intrathecal injections of methotrexate, cytarabine, and dexamethasone to prevent central nervous system leukemia. If the bone marrow was in remission, the patient was offered the choice to undergo bridge hematopoietic stem cell transplantation (HSCT) or continue with monoclonal antibody treatment or a traditional chemotherapy regimen. Of these, patients who opt for transplantation need to undergo HSCT bridging as soon as possible after remission to improve their prognosis. The main types of transplantation include bone marrow combine peripheral stem cells, peripheral stem cells, and umbilical cord blood stem cells. HLA matching ranges from 5/10 to 10/10.

### 2.3. Disease-related parameters

Bone marrow CR (12) was characterized by the following: (1) no clinical symptoms and signs of leukemic cell infiltration, normal or nearly normal life; and (2) hematography, defined as hemoglobin (Hb) levels ≥90 g/L, neutrophil absolute value ≥1.5 × 10^9^/L, and platelet levels ≥100 × 10^9^/L. There were no leukemic cells in the peripheral blood leukocyte classification, and (3) bone marrow: primary lymphocytes + naïve lymphocytes + naive lymphocytes ≤5%. Minimal residual lesion (MRD) negativity was defined as a positive screening marker count of <1 × 10^−4^ on flow cytometry (instrument: BDCANTO II) monitoring the immunotyping of leukemia at initial diagnosis/recurrence (13). High-risk cytogenetic subsets were defined as: (1) MRD ≥1% in patients with non-hyperdiploid B-ALL at day 46; (2) high diploid B-ALL patients had MRD ≥1% at day 46 and remained positive (≥0.01%) after two consolidation treatments (HD-MTX); (3) MRD ≥0.01% occurred at any time in intermediate risk patients during interphase treatment and was confirmed as; (4) MLLr-ALL, age <6 years, and WBC ≥300 × 10^9^/L; (5) TCF3-HLF/t(17;19).

### 2.4. Safety assessment

In terms of safety assessment, the grading of cytokine release syndrome (CRS) followed the grading system proposed by Lee et al. (14). Immune effector cell-associated neurotoxicity syndrome (ICANS) is defined according to the American Society for Bone and Marrow Transplantation consensus grading criteria (15). Other adverse events were graded according to the Common Terminology Criteria for Adverse Events version 5.0 (16), and the GVHD grading criteria were referenced from the 1994 Consensus Conference on Acute GVHD Grading (17).

### 2.5. Implantation criteria

A peripheral blood neutrophil count ≥0.5 × 10^9^/L for 3 consecutive days was considered as neutrophil implantation, and platelet count ≥20 × 10^9^/L for 7 consecutive days in the absence of platelet transfusion was considered platelet implantation (18).

### 2.6. Treatment outcome rubric (12)

Overall survival (OS) was used to evaluate all patients entering the clinical trial from the day of commencement of blinatumomab infusion until death from any cause or the date of final survival follow-up. The disease-free survival rate (DFS) was used to evaluate only patients who achieved CR, calculated from the day of commencement of blinatumomab infusion to the date of death from all causes, relapse, or the last survival follow-up.

### 2.7. Statistical analysis

SPSS 26.0 (IBM Corp., Armonk, NY, USA) statistical software was used to process the data. Count data were expressed as sample size or percentage (%). Normally distributed data were expressed as means and standard deviations. Non-normally distributed data were expressed as medians (P25–P75). OS and DFS were analyzed using Kaplan–Meier curves and log-rank tests, and the results were plotted using GraphPad Prism 8.0.2 software. Statistical significance was set at *p* < 0.05.

## 3. Results

### 3.1. Baseline characteristics

Thirty-three children with R/R BCP-ALL aged 7.6 ± 3.7 years, including 21 male (63.6%) and 11 female (36.4%) patients were treated with a short course of blinatumomab in the Children's Hospital of Soochow University between August 2021 and November 2022. Before undergoing a short course of blinatumomab treatment, the baseline levels of problastocytes were <5% in 8 cases, 5%–25% in four cases, 26%–50% in four cases, and >50% in 17 cases. Eleven cases (33.3%) were classified as refractory (seven cases were only positive for fusion genes) and 22 cases (66.7%) as relapsed. Among the 33 children, 27 had received standard chemotherapy before treatment with blinatumomab, 5 had received chemotherapy and chimeric antigen receptor T cell immunotherapy (CAR-T) therapy (3 patient received chemotherapy and one time CD19/CD22 CAR-T, one received chemotherapy, one-time CD19 CAR-T and one time CD19/CD22 CAR-T, one received chemotherapy and three-time CD19/CD22 CAR-T), and one had received chemotherapy, CD19 CAR-T and HSCT (all received once). Fusion gene expression was as follows: MLL-AF4 positive in five cases, BCR-ABL-positive in four cases, TEL-AML1 positive in three cases, and P2RYB-CRLF2 positive in twocases [Table 1].

**Table 1:** Clinical features of the participants.

Characteristics	Patient [*n* (%)]
Sex	
Male	21 (63.7)
Female	11 (33.3)
Age (years)	
≤10	23 (69.7)
>10	10 (30.3)
Pro-juvenile cell ratio before blinatumomab	
>50	17 (51.6)
26–50	4 (12.1)
5–25	4 (12.1)
<5	8 (24.2)
Refractory/Relapse	
Refractory	11 (33.3)
Relapse	22 (66.7)
Replace site	
Marrow	18 (54.6)
Marrow + extramedullary	4 (12.1)
Pre-blinatumomab therapy	
Chemotherapy	27 (81.8)
Chemotherapy + CAR-T	5 (15.2)
Chemotherapy + CAR-T + HSCT	1 (3.0)
Fusion gene	
MLL-AF4	5 (15.2)
BCR-ABL	4 (12.1)
TEL-AML1	3 (9.1)
P2RYB-CRLF2	2 (6.1)

### 3.2. Analysis of the efficacy and survival of short-course blinatumomab

**Figure 2:**
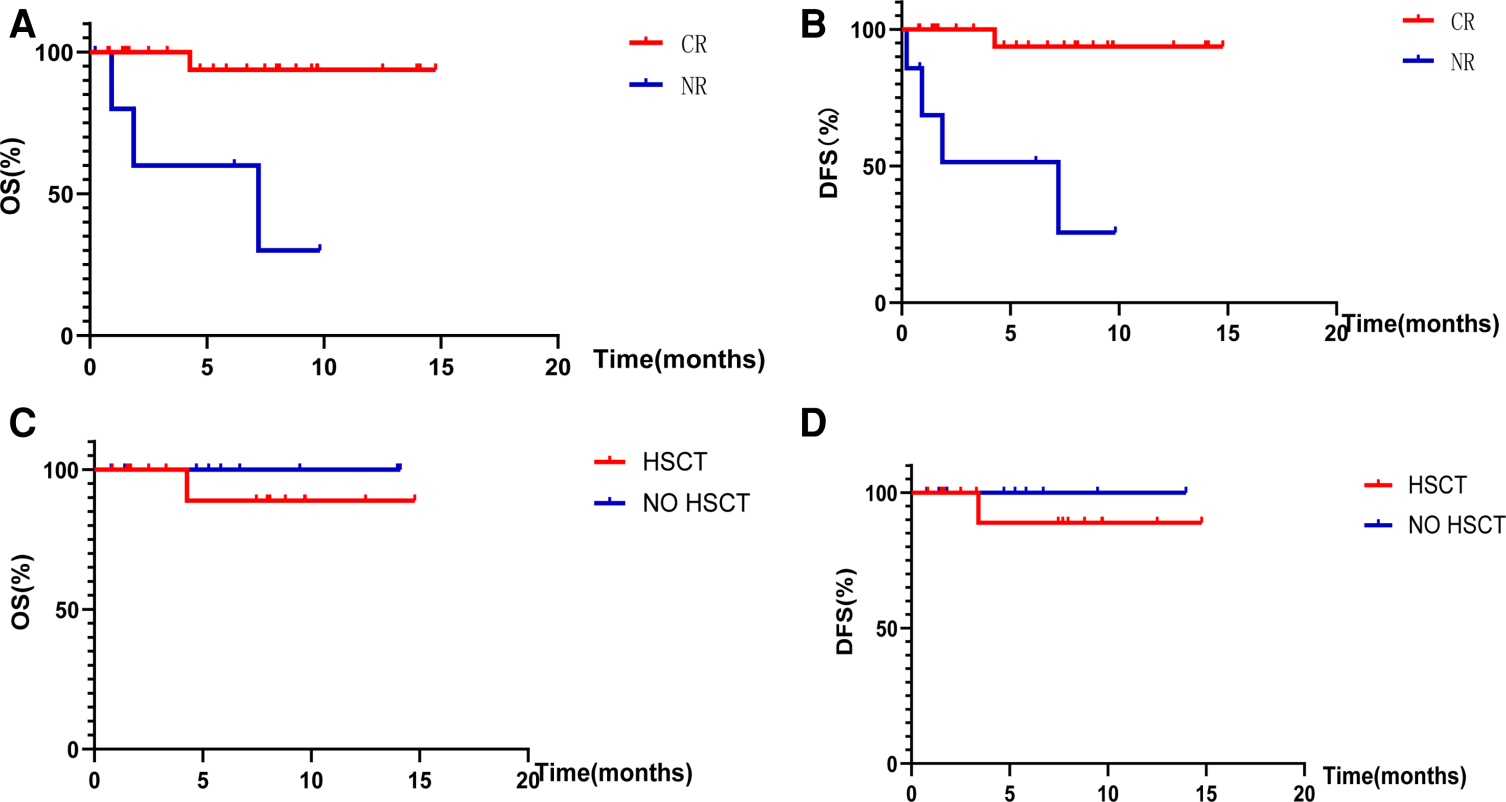
Overall survival rates and disease-free survival rates. (**A**) Shows the OS of children with CR and NR after treatment with blinatumomab. (**B**) Shows the DFS of children with CR and NR after treatment with blinatumomab. (**C**) Shows the OS of children with or without HSCT after bone marrow CR. (**D**) Shows the DFS of children with or without HSCT after bone marrow CR.

The remission rate was 78.8% (26/33) in the 33 children included in this study. Among the 26 children with CR, 15 (refractory 5, recurrent 10) underwent bridging HSCT, 8 (refractory 5, recurrent 3) continued chemotherapy owing to failure of the traditional standard chemotherapy regimen, 2 (refractory 1, relapsed 1) considered that various related adverse events would occur after transplantation and underwent a second cycle of blinatumomab therapy, and 1 (relapsed) received CAR-T therapy after CR treatment. Among the seven children who did not achieve CR, five received CAR-T cells and two discontinued treatment [Table 2]. The median follow-up time to the time of reporting was 10.7 (7.2–14.3) months. The total OS was 87.9% (28/33) and the DFS was 84.8% (28/33). The total OS of the 26 children with CR was 96.1% (25/26), and that of the seven children without CR was 57.1% (4/7). This difference was statistically significant (*p* = 0.002) [Figure 2A]. The total DFS of the 26 children with CR was 96.1% (25/26), and that of the seven children without CR was 42.9% (3/7), and the difference was statistically significant (*p* < 0.001) [Figure 2B].

**Table 2:** Efficacy of blinatumomab and follow-up time.

Case *N* = 33	Prior treatment	Prior blinatumomab			After blinatumomab			CR	Follow-up therapy	Live	Follow-up time (months)
		Primitive cells%	MRD%	Gene	Primitive cells%	MRD%	Gene				
1	Chemotherapy + CAR-T	49	40	–	1	0.01	–	Y	HSCT	Y	19.3
2	Chemotherapy	78	40.5	–	1	0.15	–	Y	CAR-T	Y	18.7
3	Chemotherapy	30	27	–	3	0.01	–	Y	Chemotherapy	Y	18.5
4	Chemotherapy + CAR-T	81	90.9	–	88	94.7	–	N	CAR-T	N	18
5	Chemotherapy + CAR-T	19	26.6	–	2	0.01	–	Y	HSCT	Y	17.1
6	Chemotherapy	90	92.2	–	1	0.01	–	Y	HSCT	Y	14.3
7	Chemotherapy + CAR-T	61	27.3	–	72	83.6	–	N	–	Y	15.1
8	Chemotherapy	77	62.9	–	5	2.36	–	N	CAR-T	Y	14.4
9	Chemotherapy	2	0.01	–	1	0.01	–	Y	Chemotherapy	Y	14
10	Chemotherapy	14	1.88	–	1	0.01	–	Y	HSCT	Y	14.3
11*	Chemotherapy	2	0.01	MLL-AF4	1	0.01	Negative	Y	HSCT	Y	13.4
12	Chemotherapy + CAR-T	24	7.65	–	2	0.01	–	Y	HSCT	Y	12.5
13	Chemotherapy	81	84.9	–	1	0.01	–	Y	HSCT	Y	12.7
14*	Chemotherapy	1	0.01	BCR-ABL	1	0.01	Negative	Y	HSCT	Y	12
15	Chemotherapy	2	0.09	–	2	0.01	–	Y	Chemotherapy	Y	11.3
16	Chemotherapy	83	88.9	–	91	94.2	–	N	CAR-T	Y	10.7
17	Chemotherapy	52	5.1	–	2	0.01	–	Y	Chemotherapy	Y	10.4
18*	Chemotherapy	3	0.01	TEL-AML1	1	0.01	Negative	Y	Chemotherapy	Y	9.3
19	Chemotherapy + CAR-T + HSCT	52	48.08	–	3	0.01	–	Y	Blinatumomab	Y	9.8
20	Chemotherapy	5	0.89	–	1	0.01	–	Y	HSCT	N	9.2
21	Chemotherapy	74	42.9	–	43	24.8	–	N	CART	N	8.4
22	Chemotherapy	3	1.9	–	1	0.01	–	Y	HSCT	Y	7.9
23	Chemotherapy	90	97.4	–	93	92.8	–	N	–	N	7.3
24*	Chemotherapy	3	8.1	MLL-AF4	2	0.01	Negative	Y	HSCT	Y	7.1
25	Chemotherapy	35	0.16	–	1	0.01	–	Y	HSCT	Y	6.1
26	Chemotherapy	45	46.7	–	1	0.01	–	Y	HSCT	Y	6.2
27*	Chemotherapy	3.5	0.01	BCR-ABL	2	0.01	Negative	Y	Chemotherapy	Y	6
28	Chemotherapy	30	4.17	–	1	0.01	–	Y	HSCT	Y	6
29	Chemotherapy	95	48.3	–	37	86.1	–	N	CAR-T	Y	5.4
30	Chemotherapy	90	95.5	–	0.5	0.01	–	Y	HSCT	Y	5.4
31*	Chemotherapy	4	0.06	BCR-ABL	1	0.01	Negative	Y	Chemotherapy	Y	5.4
32	Chemotherapy	70	51.4	–	1	0.01	–	Y	Blinatumomab	Y	5.3
33*	Chemotherapy	1	0.01	TAF15-ZNF384	2	0.01	Negative	Y	Chemotherapy	Y	5.4

(1) CR, bone marrow remission; (2)* Indicates positive fusion genes before blinatumomab treatment. (3) Before the treatment of blinatumomab, cases 4, 7, 12 received Chemotherapy and one time CD19/CD22 CAR-T, case 1 received Chemotherapy, one-time CD19 CAR-T and one time CD19/CD22 CAR-T, case 5 received Chemotherapy and 3 times CD19/CD22 CAR-T, case 19 received Chemotherapy, one time CD19 CAR-T and one time compatriot HLA matching 10/10 HSCT.

### 3.3. Treatment-related toxicity of short-course blinatumomab

In the present study, the most common associated toxicities were fever in 30 cases (90.9%), leukopenia in 28 cases (84.8%), decreased serum potassium levels in 15 cases (45.5%), thrombocytopenia in 14 cases (42.4%), hepatic impairment in 13 cases (39.4%), decreased serum calcium levels in 10 cases (30.3%), edema and oliguria in seven cases (21.2%), cardiotoxicity in seven cases (21.2%), coagulation abnormalities in six cases(18.2%), hypertension in 4 cases (12.1%), elevated uric acid levels in four cases (12.1%), CRS in 18 cases (54.5%), and ICANS in one case (3.0%). There were 163 adverse events, of which 79 (48.5%) were grade 3 or higher, including leukopenia (24 cases, 72.7%), fever (11 cases, 33.3%), thrombocytopenia (10 cases, 30.3%), hepatic impairment (10 cases, 30.3%), decreased serum calcium levels (five cases, 15.2%), decreased serum potassium levels (four cases, 12.1%), and CRS (two cases, 6.1%). No cases of ICANS were reported [Table 3]. These toxic side effects can be managed by achieving CR after reducing or temporarily discontinuing mAbs for 1–3 days, along with the implementation of symptomatic treatment.

**Table 3:** Adverse events during blinatumomab treatment.

Adverse event	All adverse events *n*(%)	Grade 3–4 adverse events *n*(%)
Fever	30 (90.9)	11 (33.3)
Leukopenia	28 (84.8)	24 (72.7)
Hypokalemia	15 (45.5)	4 (12.1)
Thrombocytopenia	14 (42.4)	10 (30.3)
Liver function damage	13 (39.4)	10 (30.3)
Hypocalcemia	10 (30.3)	5 (15.2)
Oliguria and edema	7 (21.2)	3 (9.1)
Cardiotoxicity	7 (21.2)	2 (6.1)
Abnormal coagulation function	6 (18.2)	3 (9.1)
Hypertension	4 (12.1)	2 (6.1)
Elevated uric acid	4 (12.1)	1 (3.0)
Rash	3 (9.1)	1 (3.0)
Abdominal pain	3 (9.1)	0 (0)
Hypotension	2 (6.1)	1 (3.0)
Pancreatitis	1 (3.0)	0 (0)
CRS	15 (54.5)	2 (6.1)
ICANS	1 (3.0)	0 (0)

### 3.4. Clinical outcomes of short-course blinatumomab bridge HSCT and adverse events

Among the 26 children with CR, 15 underwent bridging HSCT [Table 4] involving the transplantation of bone marrow + peripheral stem cells (5 cases), peripheral stem cells (8 cases), and umbilical cord blood stem cells (2 cases). The median time of bridging HSCT was 21 (19–23.5) days, the median time of neutrophil implantation was 13.1 ± 2.7 days, and the median time of platelet implantation was 15.9 ± 8.3 days. The median follow-up time was 10.7 (7.2–14.3) months. In 15 cases, OS was 87.9% (29/33, HR = 0.07, 95% CI: 0.01–0.92) and DFS was 84.8% (28/33, HR = 0.05, 95% CI: 0.01–0.54).

**Table 4:** A summary of 15 cases receiving blinatumomab as a bridge HSCT.

Number	Preparative regimen	Donor	HLA matching	Donor type	HSCT time	Neutrophile implantation	Platelet implantation	Final follow-up
1	CCNU + FLAG + BuCy + ATG	Father	5/10	BM + PT	38	+15	+18	CR
2	CCNU + CLAG + BU + ATG	Father	5/10	PT	26	+15	+12	CR
3	Flu + BUCy + ATG + CCNU	Father	5/10	BM + PT	14	+11	+9	CR
4	FLAG + BUCy	Unrelated donor	8/10	CB	21	–	–	–
	TSPA + Flu + ATG	Mother	5/10	PT	–	+13	+6	CR
5	CCNU + CLAG + BUCy + ATG	Father	6/10	BM + PT	18	+14	+10	CR
6	TBI + Cy + Flu + BU + MP	Mother	5/10	CB	21	+10	+14	CR
7	CCNU + FLAG + BuCy	Unrelated donor	9/10	CB	21	+20	+30	CR
8	CCNU + CLAG + BUCy	Unrelated donor	8/10	CB	30	+18	+37	CR
9	CCNU + CLAG + BUCy + ATG	Father	5/10	BM + PT	21	+11	+16	death
10	CCNU + CLAG + BuCy + ATG	Father	5/10	PT	22	+11	+13	CR
11	CCNC + CLAG + BUCy + ATG	Mother	6/10	PT	21	+12	+19	CR
12	CCNU + CLA + BUCy + ATG	Unrelated donor	10/10	PT	14	+11	+11	CR
13	CCNU + CLAG + BUCy + ATG	Sister	5/10	PT	25	+14	+25	CR
14	CCNU + TBI + Cy + ATG	Father	8/10	PT	19	+12	+10	CR
15	CCNU + CLAG + TBI + Cy + ATG	Father	5/10	BM + PT	19	+11	+12	CR

(1) Case 4 involved the first transfusion of unrelated umbilical cord blood stem cells +20d PB-STR < 1%; stem cell implantation failed, and a second maternal peripheral stem cell transplantation was performed. (2) CLAG, cladribine + cytarabine + recombinant human granulocyte-stimulating factor; TBI, total body irradiation; Cy, cyclophosphamide; FLAG, fludarabine + cytarabine + recombinant human granulocyte-stimulating factor; MP, melphalan; TSPA, thiotepa; CCNU, lomustine; Ara-c, cytarabine; BUCy, leucovorin + cyclophosphamide; BU, leucovorin; G-CSF, recombinant human granulocyte-stimulating factor; ATG, Rabbit anti-human thymocyte lymphocyte globulin; Flu, fludarabine; CB, cord blood; BM, bone marrow; PT, peripheral trunk. (3) Bridge transplantation time: time from the end of blinatumomab treatment to the return of stem cells.

The neutrophil and platelet implantation rates were 100%. Among the transplant-related adverse events, 10 cases (66.7%) were identified as acute GVHD, including 8 cases (53.3%) of skin rejection and 2 cases (13.3%) of intestinal rejection. Infection occurred in nine cases (60%), including three cases (20%) of infection by *Pseudomonas aeruginosa* combined with cytomegalovirus (CMV), two cases (13.3%) of infection with *Escherichia coli*, one case (6.7%) of infection with CMV, and three cases (20%) of infection with other pathogens. Nine patients (60%) had secondary hypertension, nine (60%) had myocardial damage, eight (53.3%) had liver insufficiency, four (26.6%) had peri-implantation syndrome, four (26.6%) had capillary leakage syndrome, three (20%) had hemorrhagic cystitis, and one (6.6%) had central nervous system disease. No hepatic small vein occlusion or thrombotic microangiopathy was observed [Table 5].

**Table 5:** Adverse events in HSCT.

Adverse event	*n* (%)
GVHD	10 (66.7)
Skin	8 (53.3)
Gastrointestinal tract	2 (13.3)
Infection	9 (60)
Pseudomonas aeruginosa + CMV	3 (20)
Escherichia coli	2 (13.3)
CMV	1 (6.7)
Other	3 (20)
Secondary hypertension	9 (60)
Cardiac damage	9 (60)
Hypohepatia	8 (53.3)
Periimplantation syndrome	4 (26.6)
Capillary leak syndrome	4 (26.6)
Hemorrhagic cystitis	3 (20)
CNS	1 (6.6)
VOD	0 (0)
TMA	0 (0)

In three cases, infection was caused by other pathogens, including one case of *Klebsiella aerogens*, one case of *Enterobacter cloacae* and *Acinetobacter Joni*, and one case involving *Escherichia coli*, CMV of *Bradystreptococcus, Parvovirus B19*, and CMV.

### 3.5. Survival analysis of bridged vs. unbridged HSCT with short-course blinatumomab therapy

The median follow-up time was 10.7 (7.2–14.3) months to the date of reporting; the total OS of the 15 children who achieved CR and underwent bridging HSCT was 93.3% (14/15) and DFS was 93.3% (14/15). The total OS of the 11 children who achieved CR without HSCT was 100% (11/11), and the DFS was 100% (11/11), with no significant difference in OS (*p* = 0.40) and DFS (*p* = 0.40) observed between bone marrow attainment of CR with or without bridging HSCT after the use of a short course of blinatumomab [Figures 2C,D].

### 3.6. Changes in immunoglobulin levels

**Figure 3:**
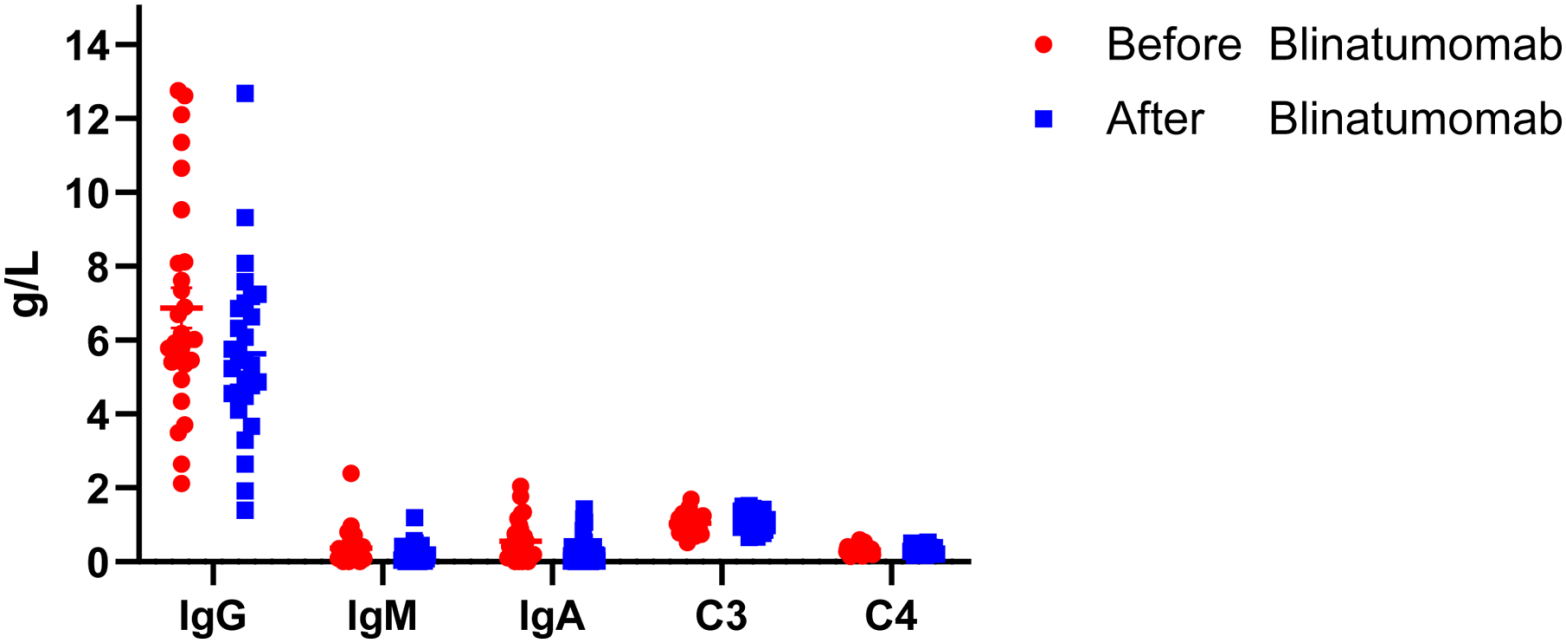
Humoral immunity levels before and after treatment in the blinatumomab.

The immunoglobulin indicators included in this study were IgG, IgM, IgA, and complement proteins C3 and C4. 5 of the 33 children had missing data, and after statistical comparison, the differences in the indicators of changes in immunoglobulin and complement protein levels before and after treatment with blinatumomab were not statistically significant in 28 children (*p* > 0.05) [Table 6, Figure 3].

**Table 6:** Comparison of humoral immune levels in blinatumomab.

Humoral immunity	*Z*	*P*
IgG	1.672	0.095
IgM	1.823	0.068
IgA	1.913	0.056
C3	0.967	0.334
C4	0.123	0.902

## 4. Discussion

Leukemia is the most common malignancy in childhood, accounting for 30% of all cancer cases in children (19). The OS and DFS of pediatric patients with ALL have improved significantly since chemotherapy-induced remission of leukemia was first proposed in 1948 (20). However, some patients remain insensitive to chemotherapy regimens, leading to relapse or refractory treatment, with long-term survival rates of only 10%–20% (21). The advent of CAR-T cell therapy has brought new hope to children with leukemia, although challenges such as difficulties in the extraction of autologous T cells in children with a high tumor burden still exist (22). Furthermore, in some cases, allogeneic CAR rejection and CAR-T cell elimination by the host immune system occur, limiting the antitumor activity of CAR-T cells (23). Both conventional and CAR T-cell therapies are subject to preparation failures and long preparation times. Furthermore, these therapies have limited effectiveness in pediatric patients with R/R BCP-ALL. In recent years, research has focused on novel approaches for the treatment of hematologic malignancies, and monoclonal antibodies have received increasing attention because they can significantly improve the remission rate of R/R BCP-ALL, improve survival outcomes, and exhibit good tolerability. Currently, blinatumomab is approved by the US FDA, European Medicines Agency, Japanese Ministry of Health, Labor, and Welfare, and the Chinese National Drug Administration for the treatment of R/R BCP-ALL in adults and children.

In a prospective multicenter phase III trial conducted by Locatelli et al., 108 children with R/R B-ALL were randomized to receive either blinatumomab (28 days) or a standard chemotherapy group with a median follow-up of 19.5 months, and OS was reported to be higher in the blinatumomab group than that in the chemotherapy group (85.2% vs. 70.4%, HR = 0.43, 95% CI: 0.18–1.01) (24). Furthermore, in the RIALTO study, 63% of the children (69/110) achieved CR within 28 days of blinatumomab treatment, with a median OS of 13.1 months (25). A phase III clinical study (the COG AALL-1331 study) by the Children's Oncology Society (COG) conducted in 155 hospitals across Europe and the United States and involving 208 children with intermediate-high-risk first relapse B-ALL and a median follow-up of 2.9 years, reported 2-year OS rates of 71.3% and 58.4% in the blinatumomab (28 days × 2 cycles) and chemotherapy groups, respectively (HR = 0.62, *p* = 0.02), and 2-year DFS rates of 54.4% and 39.0% (HR = 0.7, *p* = 0.03), respectively (26). Among the 33 children included in this study undergoing a blinatumomab treatment course of 16.3 ± 4.4 days, the response rate was 78.8% (26/33), the median follow-up time was 10.7 (7.2–14.3) months, OS was 87.9% (29/33, HR = 0.07, 95% CI: 0.01–0.92), and DFS was 84.8% (28/33, HR = 0.05, 95% CI: 0.01–0.54). The remission rate observed in this study was higher than that in the RIALTO study, and the OS and DFS rates were higher than those reported by Locatelli et al. and in the COG AALL-1331 study, which may be explained by the smaller number of included cases and shorter follow-up time in the present study. There have been studies reported, Loss of CD19 expression was associated with failure of blinatumomab therapies in 30% of the cases (27–30). While the mechanisms underlying the loss of CD19 expression are insufficiently understood (31). In conclusion, a short course of blinatumomab significantly improved the OS and DFS in children with R/R BCP-ALL.

One study reported that the commonly observed toxicities associated with blinatumomab treatment were fever (60%), headache (34%), neutropenia (28%), edema (26%), nausea (24%), hypokalemia (24%), constipation (21%), and anemia (20%), with grade 3 or higher adverse events mainly being leukopenia (38%) and lymphopenia (30%) (7, 32, 33). In seven AIEOP center studies, 22 patients (56.4%) experienced adverse events, of which 19 (41.3%) were classified as grade 3 (34). Locatelli et al. reported serious adverse event rates in 24.1% and 43.1% of patients in the blinatumomab and consolidation chemotherapy groups, respectively, with a lower incidence of grade 3 adverse events in the blinatumomab group (57.4% vs. 82.4%) than in the other groups (35). In the present study, the most common adverse events were fever, leukopenia, CRS, decreased serum potassium levels, thrombocytopenia, hepatic impairment, decreased serum calcium levels, edema, oliguria, cardiotoxicity, coagulation abnormalities, hypertension, and elevated uric acid levels. The results of this study are consistent with findings in the relevant literature. Meanwhile, some scholars believe that leukopenia and infections are not directly caused by the use of blinatumomab and may be related to the fact that the bone marrow is in a suppressed phase in children after T cell activation (36). These adverse events can be controlled and improved with aggressive symptomatic treatment. No deaths following blinatumomab-related adverse events have been reported in the medical literature, and none were observed in this study. These findings indicate that blinatumomab is a safe option for treating R/R BCP-ALL in children.

However, the need for bridging HSCT after treatment with blinatumomab remains controversial. Studies suggest that bridging HSCT after achieving CR with blinatumomab is beneficial for the long-term survival of children (37). In a study by Pawinska-Wasikowska et al., blinatumomab was used as a bridge to HSCT in 11 children who achieved a CR and then underwent HSCT (38). The median follow-up time was 24.5 (1–47 months), and all children were disease-free. In the RIALTO study, out of 110 children, 73.5% of those with CR bridged to HSCT achieved CR with a 1-year OS rate of 87% compared to the lower 1-year survival rate of 29% in children who did not undergo HSCT (HR = 0.16, 95% CI: 0.08–0.32) (39). It has also been suggested that patients who do not undergo bridging HSCT after achieving CR may experience long-term CR (40). Newman and Benani conducted a phase II clinical trial and reported that out of 15 children who achieved remission after blinatumomab treatment, six did not undergo bridging HSCT and achieved CR at a median follow-up of 33 months (41). In the present study, with a median follow-up of 6.2 months, OS was 93.3% vs. 100% (*p* = 0.40) and DFS was 93.3% vs. 100% (*p* = 0.40) in 15 cases with bridging HSCT vs. 11 cases without bridging HSCT, respectively, which is consistent with the findings of Newman and Benani. Due to the high risk of complications following HSCT, along with minor side effects following a short course of blinatumomab and the possibility of long-term remission, blinatumomab can be used as a bridge to HSCT. However, whether blinatumomab can replace HSCT as a standalone treatment option requires further investigation in further clinical studies.

Blinatumomab-bridging HSCT should be initiated promptly to improve patient outcomes after a short course of blinatumomab-induced remission. Uchida et al. reported a case in which molecular CR was achieved with localized chronic graft-vs.-host disease in a patient who underwent HSCT on the 22nd day after CR in the bone marrow (42). In another study, Chen et al. reported the outcomes of two children who underwent allogeneic HSCT within one month of blinatumomab treatment with no implant failure. One patient experienced a relapse 4 months after transplantation; however, after 14 days of blinatumomab treatment, the patient achieved CR. Another patient received maintenance therapy with flumatinib after transplantation and the condition remained stable at the time of reporting (43). In the present study, all 15 patients achieved CR before undergoing HSCT, with a median time to transplantation of 21 (19–23.5) days, which was within one month, consistent with that of the study by Chen et al. However, in the present study, patients 2 and 5 experienced relapse 53 days and 15 days after achieving bone marrow remission, respectively. Therefore, there is still the possibility of recurrence within 1 month of a short course of blinatumomab treatment for CR. Currently, there is no consensus regarding the optimal time interval for HSCTs after remission following a short course of blinatumomab therapy, although prompt transplantation is preferred.

Although our study presented some limitations in terms of its retrospective design and the limited number of children included, our results suggest that a short course of blinatumomab not only achieves bone marrow remission in children with R/R BCP-ALL but also offers hope for long-term survival with fewer side effects during treatment. In developing countries, a short course of blinatumomab can achieve satisfactory outcomes, while reducing household costs and saving medical resources. However, the necessity of bridging HSCT after achieving CR needs to be further investigated in future research.

## 5. Conclusions

Our results demonstrated that the remission rate of R/R BCP-ALL in children treated with a short course of blinatumomab was high and the toxic side effects were low and controllable. The OS and DFS of children with CR were significantly higher than those of children without CR, and no obvious short-term survival benefits were observed after a short course of blinatumomab bridging HSCT. However, owing to the small sample size included in this study and the short follow-up time, the conclusions need to be confirmed by large-scale multi-center clinical studies.

## Data Availability

The original contributions presented in the study are included in the article/Supplementary Material, further inquiries can be directed to the corresponding authors.
